# Imaging of serotonin transporters with [^123^I]FP-CIT SPECT in the human hypothalamus

**DOI:** 10.1186/2191-219X-3-34

**Published:** 2013-04-25

**Authors:** Anke J Borgers, Anneke Alkemade, Elsmarieke M Van de Giessen, Madeleine L Drent, Jan Booij, Peter H Bisschop, Eric Fliers

**Affiliations:** 1 Department of Endocrinology and Metabolism, Academic Medical Centre, University of Amsterdam, Meibergdreef 9, Room F5-168, Amsterdam, 1105 AZ, The Netherlands; 2 Department of Nuclear Medicine, Academic Medical Centre, University of Amsterdam, Amsterdam, The Netherlands; 3 Department of Internal Medicine, Section of Endocrinology, Neuroscience Campus Amsterdam, VU University Medical Centre, Amsterdam, The Netherlands

**Keywords:** Serotonin transporter imaging, [^123^I]FP-CIT, SPECT, Human, Pituitary insufficiency, Hypothalamus, SERT

## Abstract

**Background:**

Serotonergic neurons in the rodent hypothalamus are implicated in key neuroendocrine and metabolic functions, including circadian rhythmicity. However, the assessment of the serotonergic system in the human hypothalamus *in vivo* is difficult as delineation of the hypothalamus is cumbersome with conventional region-of-interest analysis. In the present study, we aimed to develop a method to visualize serotonin transporters (SERT) in the hypothalamus. Additionally, we tested the hypothesis that hypothalamic SERT binding ratios are different between patients with hypothalamic impairment (HI), pituitary insufficiency (PI), and control subjects (C).

**Methods:**

SERT availability was determined in 17 subjects (6 HI, 5 PI, and 6 healthy controls), 2 h after injection of ^123^I-*N*-ω-fluoropropyl-2β-carboxymethoxy-3β-(4-iodophenyl) nortropane ([^123^I]FP-CIT), using single-photon emission computed tomography (performed on a brain-dedicated system) fused with individual magnetic resonance imaging (MRI) scans of the brain. The hypothalamus (representing specific SERT binding) and cerebellum (representing nonspecific binding) were manually delineated on each MRI to assess [^123^I]FP-CIT binding and specific-to-nonspecific binding ratios.

**Results:**

In each healthy subject, [^123^I]FP-CIT binding was higher in the hypothalamus than in the cerebellum, and the mean hypothalamic binding ratio of SERT was 0.29 ± 0.23. We found no difference in hypothalamic binding ratios between HI, PI, and control subjects (HI 0.16 ± 0.24, PI 0.45 ± 0.39, C 0.29 ± 0.23, *p* value 0.281).

**Conclusions:**

We were able to demonstrate SERT binding in the human hypothalamus *in vivo*. However, we did not find altered hypothalamic SERT binding in patients with hypothalamic impairment.

**Trial registration:**

Netherlands Trial Register: NTR2520

## Background

The human hypothalamus is a small brain structure of only 4 ml in the diencephalon that directs a multitude of important functions in the body, including pituitary hormone release, diurnal rhythmicity, energy homeostasis, and autonomic regulation [1]. The serotonergic system is one of the key regulators of these functions [2-6]. Animal studies showed that numerous hypothalamic areas receive axon collaterals from serotonergic perikarya located in the midbrain [7,8]. Hypothalamic microinjection of serotonergic agents into brain-cannulated rats produces potent and selective effects on feeding patterns and food choice [9]. Moreover, serotonergic stimulation of selected hypothalamic areas in rodents affects energy metabolism [10], circadian rhythmicity [11], and cardiovascular responses [4]. By inference, dysfunction of the serotonergic system is likely to be one of the determinants of symptoms in patients with hypothalamic dysfunction such as obesity, disturbed sleep, and drowsiness [12-14].

Imaging of serotonin transporters (SERT) with single-photon emission computed tomography (SPECT) or positron emission tomography (PET) provides an important opportunity to study the serotonergic system *in vivo*. SERT are expressed exclusively in the membrane of serotonergic neurons and regulate intrasynaptic neurotransmitter levels. The concentration of transporters is assumed to reflect the homeostatic tone of neurotransmitter systems [15]. Several studies have investigated SERT *in vivo* in the diencephalon in humans [16-19], providing strong evidence for expression of SERT in the human diencephalon. However, the expression of SERT in the hypothalamus was poorly defined as spatial resolution of nuclear imaging techniques is limited, and delineation of a structure as small and heterogeneous as the hypothalamus is cumbersome with conventional region-of-interest (ROI) analysis [20]. To our knowledge, only one study demonstrated hypothalamic SERT binding using PET and [^11^C]DASB, although the delineation of the hypothalamus was not strictly defined [21].

The aim of this study was to evaluate whether SERT binding can be demonstrated in the human hypothalamus *in vivo* using SPECT imaging and ^123^I-*N*-ω-fluoropropyl-2β-carboxymethoxy-3β-(4-iodophenyl)nortropane ([^123^I]FP-CIT). For this purpose, we combined conventional magnetic resonance imaging (MRI) for anatomical reference with SPECT imaging of the SERT with [^123^I]FP-CIT using a brain-dedicated system [22,23]. This radiotracer is approved to visualize and quantify dopamine transporters as early as 3 h after injection [24], but more recent studies showed its capacity to assess specific binding to extrastriatal SERT as well. For instance, in rats, [^123^I]FP-CIT binding in the hypothalamus could be blocked as well as displaced by a selective serotonin reuptake inhibitor [25,26]. MDMA (a selective neurotoxic drug for serotonin neurons) was able to reduce hypothalamic binding of β-CIT (a radiotracer pharmacologically comparable to [^123^I]FP-CIT) in rats and monkeys [27]. In nonhuman primates, [^11^C]FP-CIT binding in the diencephalon was displaced by β-CIT. In humans, [^123^I]FP-CIT binding in the midbrain and diencephalon could be blocked by a selective serotonin reuptake inhibitor [22]. In addition, in an autoradiographic study of the postmortem human brain, [^125^I]β-CIT binding in the thalamus, hypothalamus, and midbrain, with the exception of the substantia nigra, could be completely displaced by addition of the selective serotonin reuptake inhibitor citalopram, indicating that binding in these areas is almost exclusive to SERT and not to dopamine transporters [28]. The capacity to assess specific binding to extrastriatal SERT in humans is optimal between 2 and 3 h after injection [23,29].

As a next step, we investigated if hypothalamic specific-to-nonspecific [^123^I]FP-CIT binding ratios are impaired in patients treated for a large sellar tumor giving rise to visual field defects. These tumors are highly suspect for giving rise to hypothalamic impairment by various factors including direct tumor invasion or involvement, trauma related to surgery, and radiation [30]. As these patients suffer from pituitary insufficiency, we included a third group with pituitary insufficiency without a history of visual field defects, radiotherapy, and surgery to correct for potential confounding by endocrine factors.

## Methods

### Subjects

Six healthy control subjects were included in the present study. Exclusion criteria were age below 18 or above 65 years; the use of medication interfering with serotonin or dopamine metabolism (e.g., psychotropic medication like SSRIs or other antidepressants); lifetime ecstasy, amphetamine, or cocaine use; intravenous drug abuse as measured by self-report; participation in another study associated with exposure to ionizing radiation during the last 12 months; pregnancy; and the presence of any contraindication for MRI. All subjects completed the Beck Depression Inventory, the Mini-Mental State Examination, the Symptoms Checklist, and Snaith-Hamilton Pleasure Scale before inclusion to exclude subjects with severe neuropsychiatric problems.

Furthermore, eligible patients with clinical suspicion of hypothalamic impairment and patients with pituitary insufficiency, i.e., at least one impaired anterior pituitary hormonal axis, were recruited from the outpatient clinic of the Department of Endocrinology and Metabolism of the Academic Medical Centre and the Department of Endocrinology of the VU Medical Centre. All patients were seen on a regular basis by an internist-endocrinologist for clinical and biochemical evaluation. They received conventional hormone replacement therapy consisting of l-thyroxin, hydrocortisone, testosterone, recombinant human growth hormone, and/or vasopressin analogues when indicated. Exclusion criteria were identical to those for healthy control subjects.

We selected three groups that were carefully matched for age and gender: (1) six healthy control subjects (C); (2) six subjects with probable hypothalamic impairment (HI), defined as having a history of surgery in the sellar region, cranial radiotherapy, as well as compression of the optic chiasm; and (3) five subjects with pituitary insufficiency (PI), without a history of cranial surgery, radiotherapy, or compression of the optic chiasm. The HI group consisted of subjects treated for non-functioning macroadenoma (*n* = 3), craniopharyngioma (*n* = 1), growth hormone (GH)-producing macroadenoma (*n* = 1), or dysgerminoma (*n* = 1). All subjects with HI were adrenocorticotropic hormone (ACTH)-, thyroid-stimulating hormone (TSH)-, and luteinizing hormone/follicle-stimulating hormone (LH/FSH)-deficient; *n* = 5 had GH deficiency, and *n* = 2 had antidiuretic hormone (ADH) deficiency. In the PI group, *n* = 3 subjects had Sheehan syndrome, and *n* = 2 subjects had pituitary apoplexy. All subjects with PI were ACTH-, GH-, and LH/FSH-deficient, and *n* = 4 were TSH-deficient. As expected, the three groups were comparable with respect to age, sex, and body mass index (Table 1). Written informed consent was obtained from all subjects, and the study was approved by the Medical Ethical Committee of the Academic Medical Centre from the University of Amsterdam and performed in accordance with the Declaration of Helsinki.

**Table 1:** Clinical characteristics

	**HI**	**PI**	**Control subjects**	***p *value**
	***n *= 6**	***n *= 5**	***n *= 6**	
Age (year)	51.0 ± 6.0	53.8 ± 6.1	49.7 ± 7.4	0.590
Male/female (*n*)	2/4	2/3	2/4	0.966
Body mass index, kg/(height)^2^	32.7 ± 10.1	29.3 ± 5.5	26.6 ± 2.0	0.333
ACTH deficiency, *n* (%)	6 (100)	5 (100)	0 (0)	
GH deficiency, *n* (%)	5 (83.3)	5 (100)	0 (0)	
TSH deficiency, *n* (%)	6 (100)	4 (80)	0 (0)	
LH/FSH deficiency, *n* (%)	6 (100)	5 (100)	0 (0)	
ADH deficiency, *n* (%)	2 (33.3)	0	0 (0)	

HI, patients with probable hypothalamic impairment, defined as having a history of surgery in the sellar region, cranial radiotherapy, and compression of the optic chiasm; PI, subjects with pituitary insufficiency but without a history of cranial surgery, radiotherapy, or compression of the optic chiasm.

### [^123^I]FP-CIT brain SPECT imaging

Subjects were examined using SPECT with the ligand [^123^I]FP-CIT, which has a high affinity for the dopamine transporter and somewhat lower affinity for the SERT. Radiosynthesis of [^123^I]FP-CIT was performed as described earlier [31]. To block the uptake of free radioactive iodide in the thyroid, each subject received 300 mg of potassium iodide in the 24 h before the SPECT imaging. Acquisition of the SPECT images took place at 2 h after an intravenous bolus injection of approximately 115 MBq [^123^I]FP-CIT (range, 110 to 120 MBq). They were performed using a 12-detector single-slice brain-dedicated scanner (Neurofocus 810, which is an upgrade of the Strichmann Medical Equipment, Cleveland, OH, USA) with a full-width at half-maximum resolution of approximately 6.5 mm throughout the 20-cm field of view. Subjects were positioned with their head parallel to the orbitomeatal line to acquire axial slices parallel and upward from this line to the vertex in 5-mm steps. The energy window was set at 135 to 190 keV. Attenuation correction of all images was performed as described earlier [32], and all images were reconstructed in three-dimensional (3-D) mode.

### MRI

For anatomical reference, a T1-weighted 3-D MRI scan was acquired from each individual using a 3-T Philips Intera scanner (Philips Healthcare, Best, The Netherlands) with a standard head coil.

### Image analysis

To analyze the brain SPECT images, we defined ROIs for the hypothalamus and cerebellar cortex (excluding the vermis) in each participant. These unique ROIs were manually drawn by experienced researchers in the field of the hypothalamus (AA and EF) on each individual T1-weighted 3-D MRI scan using in-house-developed software [33]. AA and EF were blinded to the clinical data. Using the same software, SPECT scans of the subjects were manually matched with their individual T1-weighted 3-D MRI scan. In the first step, the individual MRI scan was reoriented towards the anterior-posterior commissure line. Second, the individual SPECT data were overlaid onto the individual MRI and manually matched in all three (*x*, *y*, *z*) planes. Finally, the mean amounts of radioactivity/voxel were determined for each ROI. Activity in the cerebellar cortex (excluding the vermis) was assumed to represent nonspecific binding. The specific-to-nonspecific binding ratios were calculated as follows: (Binding in hypothalamus − Nonspecific binding in the cerebellar cortex) / Nonspecific binding in the cerebellar cortex [34,35].

### Delineation of the hypothalamus on MRI

**Figure 1:**
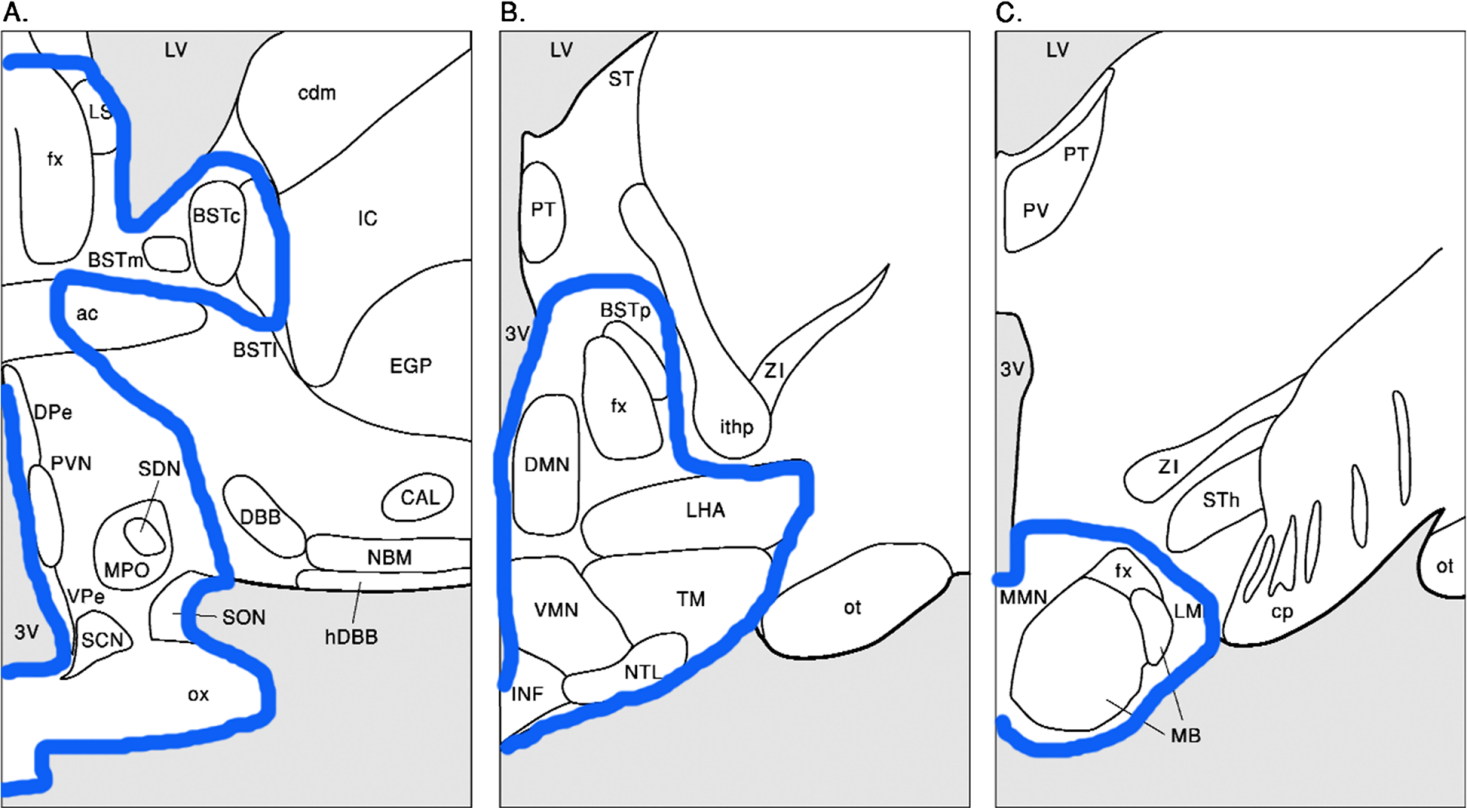
**Delineation of the hypothalamus in coronal view.** (**A**), (**B**), and (**C**) represent different levels of the hypothalamus, from rostral (**A**), middle (**B**), to caudal (**C**). Note that only one side of the hypothalamus is shown.

**Figure 2:**
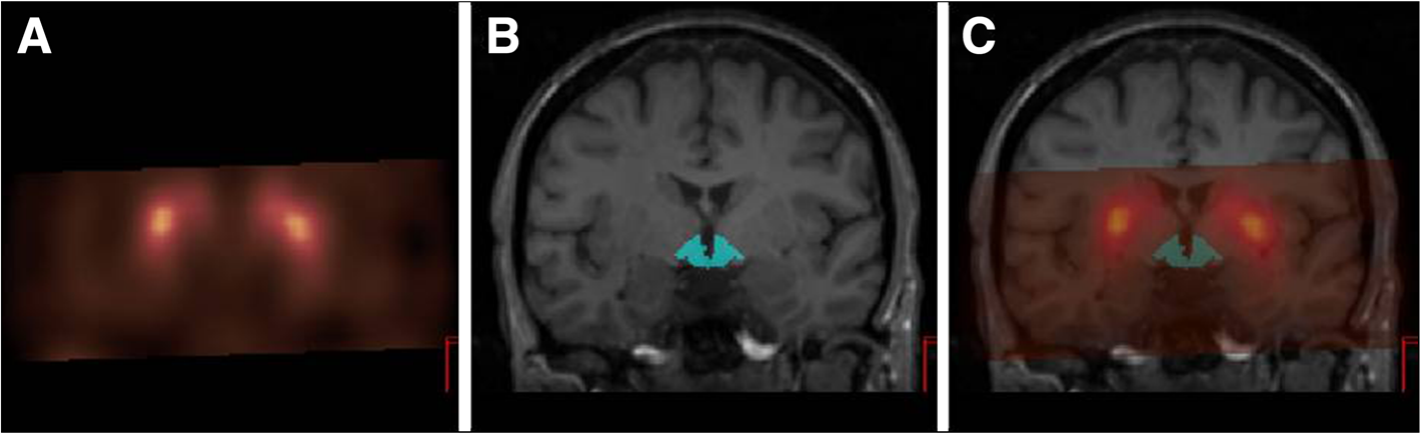
**SPECT and MRI of the hypothalamus.** (**A**) Coronal SPECT image of a healthy subject 2 h after injection of approximately 115 MBq [^123^I]FP-CIT at the level of the hypothalamus. (**B**) Coronal T1-weighted MRI image of the same subject with ROI drawn on the hypothalamus. (**C**) Coregistered SPECT and T1-weighted MRI image with ROI drawn on the hypothalamus. The SPECT images are color encoded for low (black) to high activity (yellow).

We delineated the hypothalamus as visualized schematically in Figure 1, using anatomical landmarks wherever possible. Rostral border: lamina terminalis, where the optic chiasm attaches to the mediobasal hypothalamus. Lateral border: as indicated in Figures 1 and 2. Dorsal border: septum verum. We included the area of the bed nucleus of the stria terminalis and the lateral septum. At a more caudal level (Figure 1B), we used the sulcus hypothalamicus as dorsal border. Caudal border: we included the mammillary bodies as the most caudal hypothalamic structures.

### Sample size calculation

In a previous study by Booij et al., [^123^I]FP-CIT binding ratios to SERT in the diencephalon of healthy subjects were 0.51 ± 0.17 [22]. Blocking of SERT in the diencephalon by paroxetine (a selective serotonin reuptake inhibitor) decreases this binding ratio to 0.17 ± 0.15. As no preliminary data were available regarding the effect of HI on serotonergic neurotransmission in the hypothalamus, we used these values to calculate our sample size, assuming that healthy controls will have a binding ratio of [^123^I]FP-CIT to SERT in the diencephalon of 0.51 ± 0.17, that HI patients will have a binding ratio of 0.17 ± 0.15, and that PI patients will have a binding ratio of 0.34 ± 0.16. To detect a difference between the three groups (hypothalamic impairment vs. pituitary insufficiency without hypothalamic impairment vs. healthy controls) with significance level *α* = 0.05, power = 80%, variance of means = 0.019, and a common standard deviation = 0.16, we needed six subjects per group (used software: nQuery Advisor 7.0, 1995–2007, developed by Janet D. Elashoff).

### Statistics

Statistical analysis was done using PASW Statistics for Windows, version 19.0 (SPSS Inc. Chicago, IL, USA). Numerical variables were presented as mean ± SD and categorical variables as counts (percentages). Interobserver variability in the hypothalamic specific-to-nonspecific binding ratios was assessed using intraclass correlation coefficient (ICC). Differences between the three groups were tested with one-way ANOVA or chi-square test where appropriate. A two-sided *p* value <0.05 was considered as statistically significant.

## Results

### SPECT measures of SERT in the hypothalamus in healthy control subjects

In each healthy control subject, [^123^I]FP-CIT binding was higher in the hypothalamus than in the cerebellum, and the mean hypothalamic binding ratio of SERT was 0.29 ± 0.23. We found no difference in the hypothalamic binding ratios of SERT between HI, PI, and control subjects (HI 0.16 ± 0.24, PI 0.45 ± 0.39, C 0.29 ± 0.23, *p* value 0.281). Of note, there was a very good interobserver agreement between the two independent observers (ICC 0.951, 95% confidence interval (CI) 0.872 to 0.982).

## Discussion

This study is the first to demonstrate *in vivo* SERT binding in the human hypothalamus using [^123^I]FP-CIT SPECT. We were able to demonstrate hypothalamic SERT binding by fusing SPECT scans with individual conventional MRIs. Previous SPECT studies have reported SERT binding in the human thalamus/hypothalamus region, hypothalamic/midbrain area, or diencephalon [18,22,36], and one study examined human hypothalamic SERT binding using PET imaging and [^11^C]DASB [21]. However, the delineation of the hypothalamus in those studies was not as precise as in our present method. We used 3T-MRI for anatomical reference to manually draw unique templates of each hypothalamus. The technique used overcomes the lack of anatomical reference on SPECT images, which has often been a methodological limitation. Moreover, two experts with extensive knowledge on the neuroanatomy of the human hypothalamus delineated the hypothalamus with a very good interobserver agreement. This is an important aspect as the exact borders of the hypothalamus are not a matter of clear-cut certainty [37,38], and the distribution of important cell types is not necessarily limited by classical hypothalamic neuroanatomical landmarks as visualized by Nissl staining.

The mean hypothalamic SERT binding ratio of the present study appeared to be lower than previously reported in the diencephalon [23], possibly related to a more precise delineation of the target area in the present study and to age differences. Central SERT availability declines with physiological aging [39], and our study included subjects older than those in the study by Koopman et al. [23]. Of note, the binding ratio in the group of control subjects, as well as in the HI group, is significantly above 0 (one-sample *t* test: *t* = 3.09, *p* = 0.027), and the variability in binding ratio is in line with that reported by Kupers et al. [21].

We observed a relatively large interindividual variation in SERT binding potential. This is in line with previous observations of SERT in the midbrain and diencephalon areas [21,23,40,41]. We cannot rule out having systematically underestimated the binding ratio as the cerebellum contains small amounts of SERT and due to partial volume effects [42,43]. Using the cerebellum to correct for nonspecific binding, this underestimation is expected to be 7% at most [44].

Contrary to our hypothesis, we were unable to demonstrate differences in hypothalamic SERT binding ratios between HI, PI, and control subjects, suggesting that hypothalamic serotonergic neurotransmission is not severely affected in patients suspect for hypothalamic impairment. This is remarkable as serotonin plays a very important role in the hypothalamus [2-6], which is supported by immunohistochemical studies in animals [2,45,46] and humans [47] showing strong SERT immunoreactivity in the hypothalamus. Moreover, patients treated for a sellar tumor giving rise to compression of the optic chiasm often continue to experience some physical and mental impairment despite proper endocrine substitution therapy [48-54]. Interestingly, their impairments show many similarities with the diverse functions of the hypothalamus and the serotonergic system.

In some, but not all, subjects with HI, we were able to identify anatomic hypothalamic abnormalities on the MRI scans. This did not preclude precise delineation, given the excellent interobserver agreement between the two independent observers (ICC for the HI group 0.943, 95% CI 0.567 to 0.992).

However, several limitations of our study should be mentioned. First, subjects having HI and PI are not readily available as their disease is a relatively rare condition [55]. We managed to include six patients with HI, in line with our power calculation, and matched each of them with six age- and gender-matched controls and five subjects with PI. Moreover, the power calculation was based on the effect of blocking agents on SERT availability. With our current results, the power of the study is low. We cannot exclude that the effect of hypothalamic impairment is more subtle and therefore not detectable with the current design. Furthermore, a straightforward clinical definition of HI is lacking. Subjects in the HI group were selected on the basis of a history of cranial radiation therapy, cranial surgery, and expanding tumor of the sellar region. Unfortunately, conclusive proof of hypothalamic impairment is difficult to establish as the functions of the hypothalamus are highly diverse, and validated clinical tests or imaging modalities to assess the integrity of hypothalamic function are lacking. Therefore, it is possible that some of our HI patients have only minor hypothalamic impairment which may mitigate overt serotonergic dysfunction.

## Conclusions

We were able to demonstrate SERT binding in the human hypothalamus *in vivo*. This technique will allow functional studies on hypothalamic SERT in various pathologies. In particular, our technique might be of interest for future studies on mood disorders and food intake regulation, given the importance of the serotonergic system and hypothalamus in these processes. We did not find altered specific-to-nonspecific [^123^I]FP-CIT binding ratios in patients treated for a large sellar tumor giving rise to visual field defects, although a number of methodological issues preclude a definitive conclusion.

## Competing interests

JB is a consultant at GE Healthcare. The other authors declare that they have no competing interests.

## Authors' contributions

All authors contributed substantially to the scientific process leading to this manuscript. Authors AA, AJB, EF, JB, and PHB contributed to the concept and design of the study. AJB and EMG acquired data on the subjects. AA, AJB, and EF analyzed the data. AJB drafted the manuscript, which was revised by EF and PHB. AA, EMG, JB, and MLD critically contributed to the manuscript. All authors read and approved the final manuscript.
